# Frontal fibrosing alopecia: A review of disease pathogenesis

**DOI:** 10.3389/fmed.2022.911944

**Published:** 2022-07-25

**Authors:** Yu-Jie Miao, Jing Jing, Xu-Feng Du, Mei-Qi Mao, Xiao-Shuang Yang, Zhong-Fa Lv

**Affiliations:** ^1^Department of Dermatology, Second Affiliated Hospital, School of Medicine, Zhejiang University, Hangzhou, China; ^2^Department of Dermatology, Wuxi People’s Hospital, Nanjing Medical University, Wuxi, China

**Keywords:** frontal fibrosing alopecia, immune privilege collapse, epithelial-mesenchymal transition, neurogenic inflammation, pathogenesis

## Abstract

Frontal fibrosing alopecia (FFA) is a primary patterned cicatricial alopecia that mostly affects postmenopausal women and causes frontotemporal hairline regression and eyebrow loss. Although the incidence of FFA has increased worldwide over the last decade, its etiology and pathology are still unclear. We cover the latest findings on its pathophysiology, including immunomodulation, neurogenic inflammation, and genetic regulation, to provide more alternatives for current clinical treatment. A persistent inflammatory response and immune privilege (IP) collapse develop and lead to epithelial hair follicle stem cells (eHFSCs) destruction and epithelial-mesenchymal transition (EMT) in the bulge area, which is the key process in FFA pathogenesis. Eventually, fibrous tissue replaces normal epithelial tissue and fills the entire hair follicle (HF). In addition, some familial reports and genome-wide association studies suggest a genetic susceptibility or epigenetic mechanism for the onset of FFA. The incidence of FFA increases sharply in postmenopausal women, and many FFA patients also suffer from female pattern hair loss in clinical observation, which suggests a potential association between FFA and steroid hormones. Sun exposure and topical allergens may also be triggers of FFA, but this conjecture has not been proven. More evidence and cohort studies are needed to help us understand the pathogenesis of this disease.

## Graphical Abstract

Different hypothetical triggers, such as facial soap, cosmetics, sunscreen, or stressful conditions, can cause FFA in potentially genetically susceptible individuals. PPAR-γ/TGF-β pathway plays an essential role in EMT and fibrosis. mTOR signaling works as a modulator of PPAR-γ activity and lipid homeostasis. Arrows and lines indicate a regular relationship between two elements mTOR: mammalian target of rapamycin; PPAR-γ: peroxisome proliferator-activated receptor γ; DHEA: dehydroepiandrosterone; HF: hair follicle; IFN: interferon; TGF-β: transforming growth factor-β; IP: immune privilege; EMT: epithelial-mesenchymal transition; SP: substance P; NK-1: Neurokinin-1. Created with BioRender.com (accessed on 7 February 2022). 
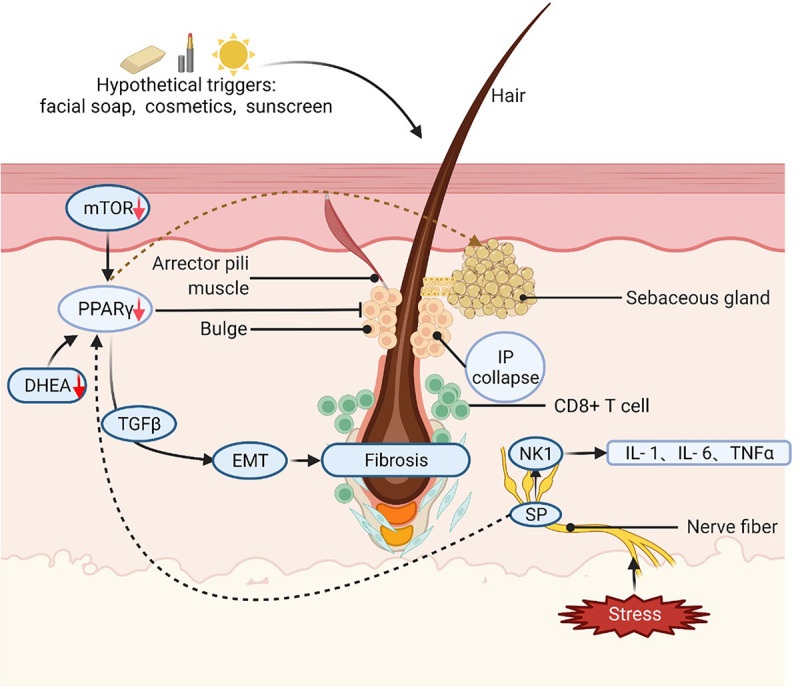

## Introduction

Frontal fibrosing alopecia (FFA) is a type of primary lymphocytic cicatricial alopecia that leads to irreversible alopecia. In 1997, Kossard described FFA as an uncommon frontal variant of lichen planopilaris (LPP) (1). The incidence of this disease has increased worldwide since Kossard first described it in 1994 (2). Previously, the disease was usually reported in postmenopausal women. The average age of diagnosis of FFA is approximately 56 years. However, an increase in premenopausal women with FFA was observed in the same survey, and an earlier age of onset was found in some sporadic cases (3). With the advent of global aging, we need to improve our understanding of this cicatricial alopecia to address its potential threat to human health.

**FIGURE 1:**
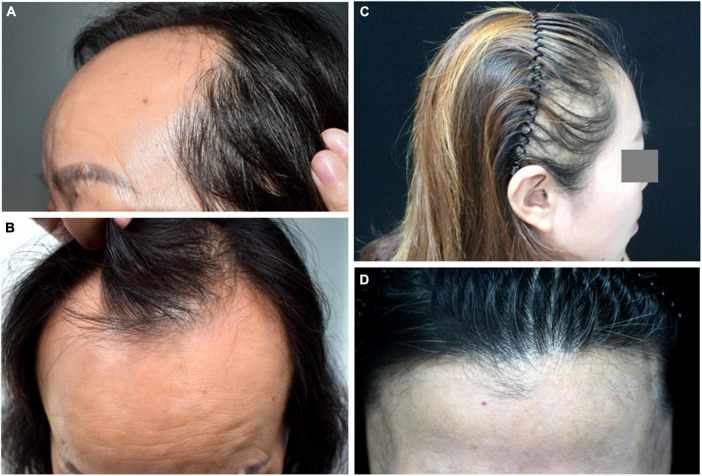
Clinical manifestation of frontal fibrosing alopecia (FFA). **(A,B)** Pattern I of FFA. **(C)** Pattern II of FFA. **(D)** Pattern III of FFA, the pseudofringe sign.

Frontal fibrosing alopecia (FFA) is a systemic skin disease with a variety of clinical manifestations and characterized by receding frontal and temporoparietal hairlines (3). The receding hairline of FFA has specific pattern characteristics and other common symptoms, such as facial papules due to vellus hair involvement. The alopecia region looks like a gleaming, atrophic, pale band of hair loss (4). Hairline recession is usually bilateral and symmetric, but some reports have described asymmetric forms. Frontal fibrosing alopecia has been classified into three clinical patterns based on frontal hairline recession (Figure 1 and Table 1) (5).

**TABLE 1:** Typical patterns of frontal fibrosing alopecia (FFA).

Pattern	Manifestation
Pattern I	Hairline recession that is linear and consistent, and behind the new hairline, there is little decrease in hair density
Pattern II	Diffuse hair loss behind the hairline with reduced hair density
Pattern III	The pseudo “fringe sign,” formed by an undamaged original frontal hairline followed by an alopecia band. It is important to note that there’s no alopecia in the brows

The slow progression of FFA results in inaccuracies in estimating the age of onset, leading to errors and interference in clinical retrospective analysis. Currently, there are few reports on the pathogenesis of FFA. We reviewed the current literature for immunomodulation, neurogenic inflammation, hormonal and metabolism, genetic and external factors to help clinicians better understand and explore the pathogenesis of this disease.

## Immunomodulation

Many reports have revealed the role of immune cell and substance-mediated inflammatory responses in the pathogenesis of FFA (6). The current findings suggest that several major immune-mediated and inflammatory mechanisms are involved in the development of FFA/LPP.

### Inflammation and immune privilege collapse

**FIGURE 2:**
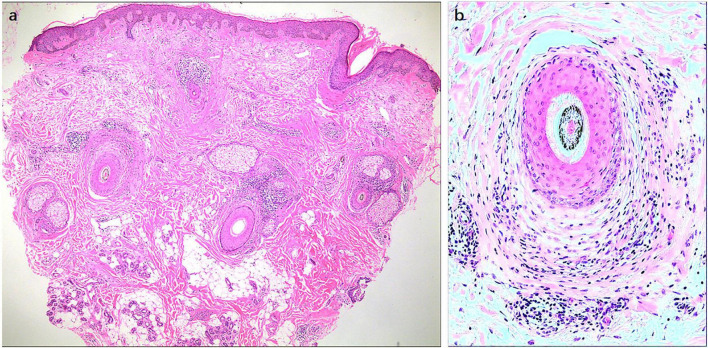
Histological findings of frontal fibrosing alopecia (FFA). Histological findings in FFA show the inflammatory cell infiltration around lesional LPP HFs, especially near the bulge area and infundibulum. Original magnification: **(a)** 40X, **(b)** 100X.

Histological findings in FFA show inflammatory cell infiltration around lesioned LPP hair follicles (HFs), especially near the bulge area and infundibulum (Figure 2), which is characterized by a rise in CD8 + cytotoxic T cells and plasmacytoid dendritic cells (6). Physiologically, epithelial hair follicle stem cells (eHFSCs) play an essential role in regulating the hair follicle cycle and entering the growth phase. HF immunity protects eHFSCs from potential autoimmune responses by downregulating the MHC class I and class II pathways (7, 8). Anagen hair bulbs are one of the few immunologically privileged tissues in the mammalian body, with negative MHC class I pathways and an immunosuppressive microenvironment. Compared to normal HFs, the expression of key markers for bulge IP maintenance, TGFβ2 and CD200 expression is reduced, whereas the main indications of IP collapse are upregulated in inflammatory lesions of LPP HFs, such as MHC class I and II β2-microglobulin (6).

Moreover, LPP has shown a reduction in Ki-67 labeling and downregulation of keratin 15, a marker of hair follicle epithelial progenitor cells in the bulge area (9, 10). Previous studies have suggested that interferon-γ (IFN-γ) may play an essential role in inducing IP collapse in HF bulge area and the ensuing damage of eHFSCs caused by the immune system (6, 11). This causes the entire follicle unit to become progressively fibrotic and unable to regenerate completely. However, it is unclear what triggers these inflammatory processes, and whether IP collapse is a major event before the inflammatory response or an extra reaction after the inflammatory response.

### Epithelial-mesenchymal transition

**Epithelial-mesenchymal transition (EMT)** is considered a normal component of embryogenesis, tissue reconstruction and wound healing, but it also occurs in pathological processes, including epithelial cell malignant transformation, cancer metastasis, and other fibrosis-related illnesses (12, 13). The loss and destruction of eHFSCs alone is not sufficient to explain the typical appearance of associated scarring. In fact, when eHFSCs were selectively ablated in mice, the resulting hair loss was not accompanied by scarring (14). The EMT marker Snail1 is positive in the dermis of lesions in FFA, enhancing the idea that EMT is involved in the pathogenesis of FFA (15, 16). Moreover, there are fibroblastoid cells within the lesional bulge epithelium and putative collagen filaments within the cytoplasm of bulge keratinocytes. Lesional FFA HFs show cells within the bulge epithelium that are abnormally positive for EMT markers, such as Snail1, Snail2, Zeb1 and TWIST1, mesenchymal markers, vimentin and fibronectin, and a cadherin switch (downregulation of E-cadherin and upregulation of N-cadherin) (17, 18).

### Peroxisome proliferator-activated receptor γ and mammalian target of rapamycin pathway

peroxisome proliferator-activated receptor γ (PPAR-γ) is involved in lipid metabolism and the differentiation and maturation of sebocytes (19). The reduction of its activity causes fibrosis (10). Karnik and his colleagues first proposed the concept of PPAR-γ and lipid metabolism in the pathogenesis of LPP. By monitoring the genetic and metabolic levels of LPP patients, they observed disturbances in fatty acid metabolism and a decrease in peroxisome and cholesterol production, which likely contributed to the accumulation of proinflammatory lipids in the patient’s hair follicles, ultimately leading to the infiltration of inflammatory cells in the bulge area (20). Previously, a new PPAR-γ modulator, N-acetyl-GED-0507-34-Levo (NAGED), was shown to not only reduce the invasion attack of inflammation on eminence but also partially restore the immune privilege of eminence (21). It also partially shows the effect of reversing the phenotype of EMT after induction of EMT *in vitro* (22, 23). PPAR-γ plays such an extensive role in the pathogenesis of FFA that we have to dedicate more attention to it.

A previous study revealed that PPAR-γ signaling was critical for the survival of eHFSCs in mice, suggesting that the loss of this receptor targeted by the keratin 15 (K15) promoter in these stem cells leads to cicatricial alopecia-like manifestations (20). However, a gene expression investigation using laser capture microdissection revealed no significant differences in PPAR expression between lesional and non-lesional bulge epithelium in the same LPP patient, which suggests that we need to find some predisposing factors in the eHFSCs region or signals other than PPAR-γ (23).

Due to the unique role of PPAR-γ in fat metabolism and hair follicle growth, another pathway, the mammalian target of rapamycin (mTOR) signaling pathway, also plays a role in these two pathways. The mTOR signaling pathway plays various roles in the immune system and stem cell proliferation (24). Previous studies have revealed that mTOR signaling works as a modulator of PPAR-γ activity and lipid homeostasis. A possible mechanism for this regulatory effect on PPAR-γ is that mTOR plays a role in the maximal transcriptional activity of a subset of PPAR-γ genes (25). A recent study showed that the PPAR-γ/mTOR signaling pathway in microglia inhibits tumor necrosis factor-α (TNF-α) and interleukin-β (IL-β) expression (26). Moreover, immunohistochemical assessment of scalp samples from patients with FFA/LPP showed that the expression of all proteins of the mTOR signaling pathway was decreased in the lesional epidermis of patients (24). More studies are needed to determine how the mTOR and PPAR-γ pathways, alone or in combination, contribute to the pathogenesis of FFA.

The PPAR-γ pathway can also suppress EMT, another critical part of FFA pathogenesis. A previous study successfully modeled EMT using a group of EMT-promoting agents in healthy human eHFSCs in full-length HF organ culture *ex vivo*. In this model, PPAR–γ agonists have been found to inhibit or even reverse EMT to some extent (18, 27) and have an antifibrotic effect in mouse models of systemic sclerosis by reducing transforming growth factor-β (TGF-β), which promotes the formation of collagen (28–31). TGF-β has multiple functions, such as driving inflammation, fibrosis, and cell differentiation. A recent report indicated that PPAR-γ activation in TGF-β transgenic mice inhibits the TGFβ-STAT3 and TGFβ-EGR1 transcriptional activation pathways in some fibrotic diseases, providing strong support for the role of the PPAR-γ/TGF-β pathway in FFA (32).

## Neurogenic inflammation

Psycho-emotional stress has been suggested as a trigger of follicle inflammation in FFA. It is worth noting that there have been reports of LPP/FFA development after hair transplantation (33), in which the Koebner phenomenon or the generation of a proinflammatory environment may impair the IP of hair follicles after transplantation. Experiments on mice have shown that stress stimulation can induce neurogenic inflammation around hair follicles, lead to an increase in mast cell degranulation, and produce inhibitive factors, such as substance P (SP) (34).

The role of neuropeptides in skin diseases has been demonstrated in many studies, and substantial evidence suggests that sensory neuropeptides play an essential role in the production and maintenance of the inflammatory cascade that causes chronic inflammation of the skin and some persistent symptoms in some diseases. Thus, calcitonin gene-related peptide (CGRP), which may be involved in lipid metabolism, has attracted the attention of some researchers (35). In many chronic inflammatory conditions, CGRP is elevated, and PPAR-γ is generally decreased (36, 37). Previous studies have shown that CGRP has immunosuppressive effects and helps protect eHFSCs from inflammatory factors such as INF-γ. However, in the same study, the upregulation of CGRP failed to restore IP of the HF (38).

In addition to PPAR-γ, SP induces the production and release of the proinflammatory cytokines IL-1, IL-6, and TNFα through the neurokinin (NK) 1 receptor signaling pathway. The upregulation of substance P expression around the bulge can mediate neurogenic inflammation and EMT. The expression of substance P was different between LPP/FFA lesional and non-lesional HFs (39). In general, LPP/FFA may have multiple pathogenic mechanisms associated with neurogenic inflammation, suggesting potential applications for new treatments.

## Hormones and metabolism

Due to the age of onset of FFA, it is vital to explore the role of steroid hormones in the pathogenesis of FFA. The mechanism of androgen action in hair loss has been previously described, while the exact mechanism of estrogen in hair growth and alopecia remains controversial. After menopause, circulating estrogen levels drop, and androgen levels rise. In the vast majority of reported cases, the onset of FFA occurs after menopause, but more recent cases of FFA have been reported in premenopausal women and men, prompting researchers to focus on the role of high androgen levels vs. estrogen in the pathogenesis (3). A recent retrospective study of 43 premenopausal women with FFA showed no abnormal sex hormone levels (40). Menopausal age, contraceptive history, and response to hormone replacement therapy were not significantly associated with the occurrence of FFA (41).

Many women with FFA also have female pattern hair loss (FPHL), and similarly, many men with FFA have male androgenetic alopecia (MAGA). A large retrospective study of 343 women and 12 men in Spain showed that 40% of affected women had concurrent FPHL and 67% of affected men had concurrent MAGA. FPHL/MAGA is a non-cicatricial alopecia disorder that is androgen-dependent in genetically susceptible individuals (42). Androgen deficiency was found in 32 of 168 FFA patients in a study specifically focused on hormonal and endocrine dysfunction (43). Dehydroepiandrosterone (DHEA) plays an essential role in androgen and estrogen biosynthesis, possesses the function of regulating PPAR and has also been found to have strong antifibrotic effects (44). Thus, downregulation of DHEA and androgens may lead to a profibrotic state of FFA. A study involving 30 female FFA patients and 34 healthy controls showed that DHEAS and androstenedione serum levels were significantly lower in FFA patients than in healthy controls (45). These studies raise the question of whether 5α reductase inhibitors are effective in treating FFA. The efficacy of 5α reductase inhibitors may be exaggerated due to the presence of FPHL/MAGA in many cases (46, 47). Therefore, it is not clear whether and exactly how 5α reductase inhibitor therapy is beneficial to FFA.

## Genetic

In previous studies, we could not establish a genetic link to FFA, although there were some reports of familial clustering in FFA patients. In 2019, Christos Tziotzios et al. conducted a genome-wide association study that found associations with FFA at four genomic loci: 2p22.2, 6P21.1, 8Q24.22, and 15Q2.1. Within loci 6P21.1 and 2p22.1, fine mapping showed associations with the HLA-B*07:02 allele and a presumed casual missense variant in CYP1B1, encoding homologous heterospecifics and hormone-processing enzymes. CYP1B1 is a widely expressed gene that codes for the cytochrome P450 1B1 microsomal enzyme (also known as exogenous monooxygenase and aromatic hydrocarbon hydroxylase). This enzyme participates in the oxidative metabolism of estradiol and estrogen into the corresponding hydroxylated catechol estrogen. Therefore, it can be speculated that increased female exposure to CYP1B1 substrates may have an active role in the development of FFA (48).

Human leucocyte antigen (HLA) profiling of a familial cluster (7 FFA members and 4 unaffected members) and 7 sporadic cases revealed two susceptible haplotypes in familial cases (C*17:01:01:02/B*42:01:01 and C*07:02:01:03/B*07:02:01:01), and three unaffected family members also had the haplotype (49). This provides us with a possible pathogenesis theory, namely, the incidence of the disease in families may indicate exposure to a common environmental trigger, possibly enhanced by hereditary susceptibility.

## Environmental factors

In the past 10–15 years, the incidence of FFA has increased year by year. It remains to be seen whether this increase is related to the improvement of the diagnosis level of dermatologists and the gradual increase in understanding of the disease. The rising incidence has suggested that FFA may be related to some exposure factors, similar to basic chronic diseases such as hypertension and diabetes. The relation of Frontal fibrosing alopecia (FFA) with environmental factors is still controversial. A recent multicentre case–control study used the odds ratio (OR) to express the effect size of different factors. It showed that FFA and formalin (OR 3.19), the use of ordinary (non-dermatological) facial soap (OR 2.09) and cream (OR 1.99), thyroid disease (OR 1.69), and alcohol scum nose (OR 2.08) are related. Smokers (OR 0.33) and anti-residue/cleaning shampoo users (OR 0.35) were negatively correlated with FFA, and FFA was not correlated with sunscreens use (50). This study reminds us to some extent that exogenous factors and the pathogenesis of FFA may have a mechanism that is still unclear.

Due to a proven correlation between LPP and light exposure, daylight exposure has also been speculated to be an environmental trigger for FFA. One hypothesis is that the light protection provided by many facial care products reduces the synthesis of the tryptophan light product 6-formylindolo [3,2-b] carbazole (FICZ), which is a potent endogenous ligand of AHR, from the photooxidation of the essential amino acid L-tryptophan. It shows proinflammatory effects at lower concentrations and anti-inflammatory effects at higher concentrations (51). However, there is no definitive evidence of a causal relationship between sunscreens or environmental factors and FFA. Relevant theories and hypotheses need to be proven by experiments and data.

## Conclusion

Frontal fibrosing alopecia (FFA) is a type of primary cicatrical alopecia. At present, many studies have shown that FFA is associated with many autoimmune diseases, further illustrating the role of complex immune regulatory networks in the pathogenesis of the disease.

From a clinical and pathological point of view, FFA is a regional disease dominated by localized eHFSC pathology as previous study have previously described (5). However, from the perspective of pathogenesis and genomics, FFA is essentially a systematic, genetically driven disease entity, and some factors may only affect the progression rate of FFA lesions without changing the development and course of the disease (48). From a regional skin presentation perspective, it means that optimal disease management first requires early and decisive intervention at the local skin level. But at its core, systemic treatments may be more effective and more important than any other approach if we aim to stop the progression of these disfiguring hair disorders as soon as possible (17). Fibrosis is a common endpoint of many diseases, and the phenotype of fibrosis is irreversible with current treatments, so it is foreseeable that EMT is a key target for early and aggressive therapeutic intervention before eHFSC is completely damaged (18). How to reverse the immune response in the early stage of EMT, or develop a corresponding targeting carrier for PPARγ, so that the PPARγ inhibitors can accumulate around the hair follicle, will become the future treatment direction or strategy to help us integrate the recent FFA pathobiological insights and translate them into specific therapeutic benefits. Commonly, it is reported that FFA is related to thyroid disease and some other autoimmune diseases, such as scalp discoid lupus erythematosus, vitiligo, lichen planus, lichen planus pigmentosa, and Sjögren’s syndrome (51).

Although the pathogenesis of FFA is complex and not completely clear at present, the incidence of FFA worldwide has been increasing over the past decade, and it is important to understand the pathogenesis of FFA to target the direction of future treatment. The results of the current study suggest that the complex interaction between immune-mediated neurogenic inflammation, genetics, hormones, and possible external stimuli provides strong evidence for the pathogenesis of FFA, and the lack of PPAR-γ plays a vital role in all stages of the disease. Genome-wide association analysis explains that FFA is an immune inflammatory disease with a genetic predisposition, which is driven by HLA-B*07:02. The role of sex steroids and external stimuli in FFA is derived from clinical studies of disease behavior, and the potential influence of both on the pathogenesis of FFA is speculative. More extensive studies are needed to verify the actual pathogenesis of FFA to provide more ideas and help solve this complex problem.

## Author contributions

Y-JM gathered the information and wrote the original draft. Z-FL and JJ were responsible for funding acquisition and modification. All authors are contributed to the article, participated in resources, wrote the review and edited, and approved the submitted version.
